# Myocardial Bridging in a 37-Year-Old Female Patient: A Case Report and Management Approach

**DOI:** 10.7759/cureus.96394

**Published:** 2025-11-08

**Authors:** Victory E Oparaocha, Loveth Igburuke, Odunayo Olorunfemi

**Affiliations:** 1 Internal Medicine, Avalon University School of Medicine, Mesa, USA; 2 Interventional Cardiology, Banner Casa Grande Medical Center, Casa Grande, USA

**Keywords:** atherosclerosis, coronary artery anomalies, exercise induced chest pain, intravascular ultrasound, myocardial bridging, optical coherence tomography, surgical unroofing

## Abstract

Myocardial bridging is a congenital coronary anomaly in which a segment of a coronary artery travels intramyocardially instead of on the epicardial surface. While often asymptomatic, myocardial bridging can occasionally result in ischemia or arrhythmias. We present the case of a 37-year-old woman with recurrent exertional chest pain and exercise-induced hypotension. Stress testing revealed mild anterior ischemia, and coronary angiography demonstrated a mid-left anterior descending myocardial bridge. Intravascular ultrasound confirmed extensive systolic compression with increased plaque burden at the bridged site. Medical therapy with vasodilators and beta-blockers was not tolerated due to adverse effects, and symptoms persisted. The patient underwent surgical unroofing of the bridged segment, which resulted in complete resolution of chest pain and improved exercise tolerance. This case highlights the role of advanced imaging in the diagnosis of MB and the importance of individualized management when medical therapy fails.

## Introduction

Up to one-quarter of the general population may harbor a myocardial bridge (MB), a congenital coronary anomaly in which a segment of an epicardial artery tunnels through the myocardium rather than resting on its surface [1]. While MB is often clinically insignificant, some cases may lead to angina, myocardial ischemia, arrhythmias, or even sudden cardiac death [2-3]. These observations challenge the notion of MB as a benign incidental finding and underscore its clinical significance. Despite this, MB remains an under-recognized cause of chest pain [4].

Diagnosis can be challenging because conventional coronary angiography may underestimate the dynamic compression of the tunneled segment, especially at rest [5]. The “milking effect” is often only visible under stress or provocation. Advanced modalities such as coronary CT angiography [6], intravascular ultrasound (IVUS), optical coherence tomography (OCT), and intracoronary functional assessment including fractional flow reserve (FFR) are therefore important in evaluating MB [7-8].

Management is generally conservative, with beta-blockers and non-dihydropyridine calcium channel blockers as first-line agents to reduce heart rate, prolong diastole, and alleviate ischemia [9]. Invasive strategies are reserved for refractory or high-risk cases, with surgical unroofing providing definitive relief when symptoms persist despite maximal tolerated therapy [10].

Herein we report the case of a 37-year-old woman with symptomatic MB refractory to medical management, in whom multimodal imaging identified both dynamic compression and associated atherosclerosis, ultimately necessitating surgical unroofing.

## Case presentation

**Figure 1:**
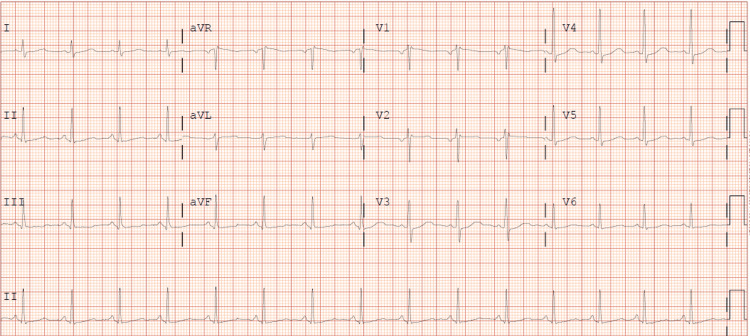
Twelve-lead ECG Twelve-lead ECG demonstrating normal sinus rhythm at rest. No ST-segment changes or arrhythmias were noted. ECG: electrocardiogram

A 37-year-old woman with no history of hypertension, dyslipidemia, diabetes, or family history of coronary artery disease presented with a two-year history of gradually progressive exertional chest pain. The pain was described as pressure-like, consistently triggered by exertion, and relieved with rest. She also experienced exercise-induced hypotension but denied associated symptoms such as nausea, diaphoresis, or syncope. Resting electrocardiogram (ECG) revealed normal sinus rhythm (Figure 1), while a stress ECG demonstrated an appropriate hemodynamic response with peak heart rate of 152 beats per minute and blood pressure response from 118/76 mmHg at rest to 158/82 mmHg at peak exercise, without significant ST-segment changes. A nuclear stress test revealed mild anterior ischemia, prompting further evaluation with coronary angiography.

**Figure 2:**
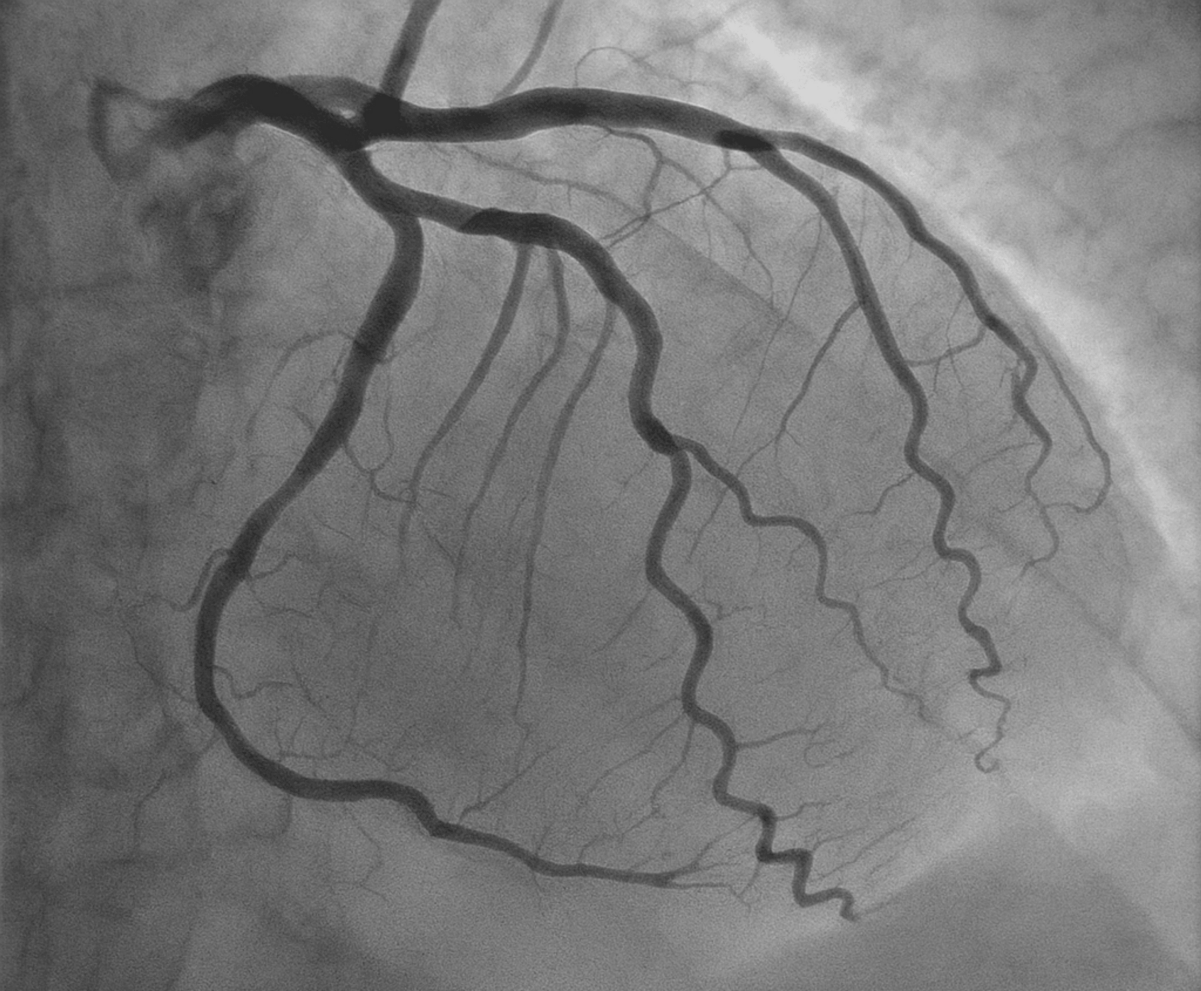
Coronary angiography projections of the LAD artery Coronary angiography projections of the LAD artery demonstrating systolic narrowing (“milking effect”) consistent with myocardial bridging. LAD: left anterior descending

Diagnostic coronary angiography (cardiac catheterization) demonstrated approximately 30% luminal narrowing of the mid-left anterior descending (LAD) artery, with angiographic evidence of myocardial bridging (Figure 2). 

The patient did not undergo coronary CT angiography prior to catheterization, as the nuclear stress test findings prompted direct invasive assessment. Intravascular ultrasound (IVUS) was performed for detailed characterization of the lesion and revealed extensive myocardial bridging involving the mid-LAD segment. The bridged segment demonstrated approximately 55% systolic compression and a slit-like appearance during systole, with minimal luminal change in the unaffected segment. There was also a localized increase in atherosclerotic plaque burden at the bridged site that was not visualized in the adjacent unaffected vessel segments. The patient was initially managed medically with a calcium channel blocker (nifedipine) and a long-acting nitrate (isosorbide mononitrate). However, she developed significant adverse effects, including symptomatic hypotension and severe headaches, leading to discontinuation of medical therapy. Beta-blocker therapy was considered but deferred due to baseline low blood pressure and poor tolerance to vasodilators. Given persistent exertional symptoms and inability to tolerate optimal medical therapy, the patient underwent surgical unroofing of the LAD MB. The operation was uneventful, and no perioperative complications occurred.

**Figure 3:**
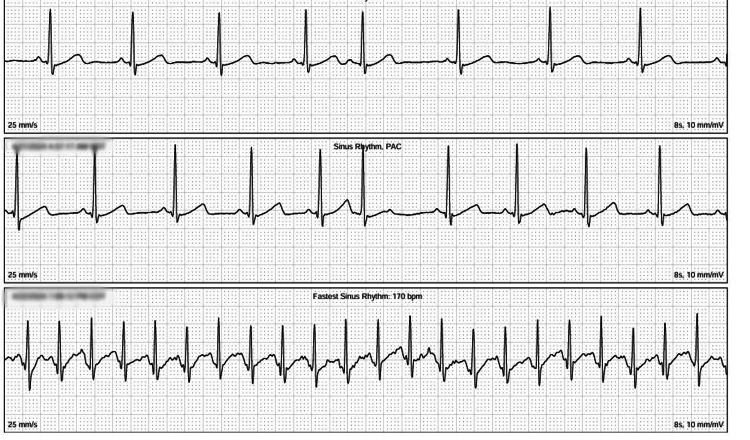
Holter monitor tracings Holter monitor tracings obtained postoperatively demonstrating sinus tachycardia without evidence of arrhythmia.

Postoperative recovery was smooth, and the patient reported complete resolution of exertional chest pain with marked improvement in exercise tolerance. Holter monitoring was performed after surgery (Figure 3) demonstrated only sinus tachycardia without any arrhythmia. She was followed clinically and with repeat exercise testing for one year postoperatively and remained symptom-free, confirming the durability of the surgical outcome.

## Discussion

Myocardial bridging is a congenital coronary anomaly most frequently involving the LAD artery. Although often considered a benign anatomical variant, MB can, in certain individuals, lead to myocardial ischemia, arrhythmias, or even sudden cardiac death [11]. The reported prevalence of MB varies widely depending on the diagnostic method used. Angiographic studies estimate 4-5%, while autopsy and CT-based imaging studies report rates approaching 20-25% [12]. Recent imaging reviews using modern CT and IVUS/OCT techniques have reaffirmed these findings and highlighted that most MBs are incidental, while only a small fraction are symptomatic [13]. Importantly, only a small subset (estimated 1-5%) of these cases are clinically symptomatic, underscoring the distinction between incidental and clinically significant MB [5-6]. High-risk MB features include deep or long tunneled segments, marked systolic compression (>50%), inducible ischemia, ventricular tachycardia, and persistent angina refractory to medical therapy [8-9]. These parameters are critical for identifying patients who may require invasive or surgical management.

Conventional coronary angiography remains a cornerstone for MB detection, but it frequently underestimates its true prevalence because the characteristic “milking effect” may not be evident at rest. CT coronary angiography (CCTA) provides high-resolution anatomical detail but is limited in assessing functional severity and dynamic changes across the cardiac cycle [5-6]. Intravascular ultrasound (IVUS), used in this case, offers real-time cross-sectional imaging that can characterize the depth and length of the bridged segment, detect systolic compression, and visualize plaque morphology [8-11]. Optical coherence tomography (OCT), with its higher spatial resolution, further delineates the intimal and medial layers, identifies microdissections or neointimal thickening, and can reveal early plaque features not seen on IVUS. Fractional flow reserve (FFR) provides a complementary physiological assessment by quantifying the hemodynamic significance of the lesion during pharmacologic provocation [7-8]. IVUS was chosen in this case because it allowed both structural and pathological assessment of the LAD, identifying atherosclerosis within the bridged segment and quantifying the degree of systolic compression, findings that were not possible with angiography or stress testing alone.

The pathophysiology of ischemia in MB is multifactorial. Systolic compression can extend into early diastole, reducing coronary perfusion, especially during tachycardia when diastolic filling time shortens [8-9]. Hemodynamic changes promote endothelial dysfunction and altered wall shear stress, particularly in the segment proximal to the bridge, predisposing to atherosclerosis [11]. Interestingly, while previous literature has consistently shown that the tunneled segment is usually spared from atherosclerosis due to protective shear forces, our patient demonstrated increased plaque formation at the bridged site itself. This finding is relatively uncommon and highlights that local atherogenesis can occasionally occur within the intramyocardial segment, likely related to variations in compression dynamics or endothelial stress.

Management of MB is usually conservative. Beta-blockers and non-dihydropyridine calcium channel blockers remain first-line therapies, reducing myocardial oxygen demand by lowering heart rate and prolonging diastole [8-9]. Vasodilators, particularly nitrates, are avoided as they may exacerbate systolic compression by reducing vascular tone [9]. When symptoms persist despite optimized therapy, invasive options are considered. Percutaneous coronary intervention (PCI) with stenting is generally avoided due to high rates of in-stent restenosis, stent fracture, and thrombosis resulting from ongoing mechanical stress in the bridged artery [9]. Surgical unroofing (myotomy) is therefore the definitive approach for medically refractory or high-risk cases. The procedure involves excising the overlying myocardial fibers to restore a normal epicardial course of the coronary artery. Although outcomes are excellent in most reports [10-14], potential complications include bleeding, coronary perforation, ventricular aneurysm, or recurrent angina due to incomplete unroofing [3-14]. Recent multicenter data confirm that contemporary unroofing carries low complication rates and durable symptom relief at long-term follow-up [14]. The one-year follow-up in our case demonstrated complete symptom resolution and sustained exercise tolerance, consistent with long-term data showing durable results after surgical myotomy [12]. This underscores the importance of individualized management, balancing medical therapy, procedural risk, and anatomical severity.

What distinguishes this case from existing literature is the identification of atherosclerotic plaque within the bridged segment rather than solely in the proximal artery, a rare finding supported by IVUS imaging. Additionally, the patient’s intolerance to both beta-blockers and vasodilators limited conventional therapy, necessitating surgical intervention. This highlights the need for flexibility in management and emphasizes the diagnostic value of multimodal imaging, particularly IVUS, in defining both structure and pathology in symptomatic MB.

Finally, while MB remains underrecognized, a practical approach for early identification can be guided by recurrent exertional chest pain in younger patients with normal coronary angiography, particularly when accompanied by exercise-induced hypotension, ischemic changes on stress testing, or unexplained angina responsive to beta-blockers [8-9]. These clinical manifestations may help physicians consider MB early and pursue confirmatory imaging when standard workups are inconclusive.

## Conclusions

This case highlights the importance of considering MB in the differential diagnosis of exertional chest pain, particularly in younger patients without traditional cardiovascular risk factors and with findings such as exercise-induced hypotension. Although MB is often incidental, a small subset of patients develop clinically significant ischemia that requires detailed diagnostic evaluation. A multimodal imaging approach combining angiography, IVUS, and stress testing is crucial for confirming the diagnosis and identifying both anatomical and functional significance. In this patient, IVUS not only quantified systolic compression but also revealed atherosclerotic plaque within the bridged segment, an uncommon finding that adds to current literature and supports the role of advanced imaging in understanding MB pathophysiology.

Medical therapy remains the cornerstone of initial management, but intolerance to vasodilators and beta-blockers, along with persistent ischemic symptoms, may necessitate surgical intervention. Surgical unroofing provided complete symptom resolution, with durable results maintained at one-year follow-up, consistent with previously reported long-term outcomes. While the procedure carries potential risks such as bleeding, coronary injury, or recurrent angina from incomplete unroofing, careful patient selection and surgical expertise can minimize complications. Ultimately, this case reinforces the importance of individualized management in MB and underscores the value of IVUS in guiding therapy. It also contributes novel insight by documenting atherosclerotic involvement within the bridged segment itself, suggesting that not all MBs are equally protective against plaque formation.
